# Changes in Lipid Profile of Keratinocytes from Rat Skin Exposed to Chronic UVA or UVB Radiation and Topical Application of Cannabidiol

**DOI:** 10.3390/antiox9121178

**Published:** 2020-11-25

**Authors:** Wojciech Łuczaj, Maria do Rosário Domingues, Pedro Domingues, Elżbieta Skrzydlewska

**Affiliations:** 1 Department of Analytical Chemistry, Medical University of Bialystok, Mickiewicza 2d, 15-222 Bialystok, Poland; elzbieta.skrzydlewska@umb.edu.pl; 2 Mass Spectrometry Center, LAQV, Department of Chemistry, Campus Universitário de Santiago, University of Aveiro, 3810-193 Aveiro, Portugal; mrd@ua.pt (M.d.R.D.); p.domingues@ua.pt (P.D.); 3 CESAM, Department of Chemistry, University of Aveiro, Campus Universitário de Santiago, 3810-193 Aveiro, Portugal

**Keywords:** cannabidiol, keratinocytes, lipidomics, phospholipids, rats, UV irradiation

## Abstract

UV radiation is a well-established environmental risk factor known to cause oxidative stress and disrupt the metabolism of keratinocyte phospholipids. Cannabidiol (CBD) is a phytocannabinoid with anti-inflammatory and antioxidant effects. In this study, we examined changes in the keratinocyte phospholipid profile from nude rat skin exposed to UVA and UVB radiation that was also treated topically with CBD. UVA and UVB radiation promoted up-regulation of phosphatidylcholines (PC), lysophosphatidylcholines (LPC), phosphatidylethanolamines (PE) and down-regulation of sphingomyelin (SM) levels and enhanced the activity of phospholipase A2 (PLA2) and sphingomyelinase (SMase). Application of CBD to the skin of control rats led to down-regulation of SM and up-regulation of SMase activity. After CBD treatment of rats irradiated with UVA or UVB, SM was up-regulated and down-regulated, respectively, while ceramide (CER) levels and SMase activity were down-regulated and up-regulated, respectively. CBD applied to the skin of UV-irradiated rats down-regulated LPC, up-regulated PE and phosphatidylserines (PS) and reduced PLA2 activity. In conclusion, up-regulation of PS may suggest that CBD inhibits their oxidative modification, while changes in the content of PE and SM may indicate a role of CBD in promoting autophagy and improving the status of the transepidermal barrier.

## 1. Introduction

UV radiations are one of the main environmental factors that cause damage to skin cells [1,2]. The cells of the epidermis are particularly exposed to UV radiation, including mainly the most abundant and exposed keratinocytes, which absorb most of the UV radiation. UV rays reaching the earth’s surface contain two types of UVB (280–320 nm) and UVA (320–400 nm) radiation, which are characterized by different energy and penetrating capacity, and also lead to various changes in the metabolism of phospholipids and ceramides (CERs) of keratinocytes [3]. These metabolic disorders are still under investigation and include changes in the composition of phospholipids, as well as the metabolic consequences of these changes. It is known that exposure of keratinocytes to UV radiation leads to an increase in the generation of reactive oxygen species (ROS), and disturbances in the antioxidant system [4]. The consequence of UV irradiation is to shift the redox imbalance towards oxidative conditions, which result in oxidative modification of keratinocyte components including proteins and lipids [5]. Oxidative stress also accompanies inflammatory processes, particularly in the case of skin diseases such as atopic dermatitis or psoriasis [6,7]. One of the most common effects of oxidative stress is the oxidation of polyunsaturated fatty acids leading to peroxidation of phospholipids [8], and the generation of products of their oxidative fragmentation and cyclization [9,10]. Recently, it was found that the level of lipid peroxidation products is significantly increased in the keratinocytes of patients with psoriasis [10]. It is important that, although UV radiation causes disturbances in the cell metabolism of epidermal phospholipids, UV phototherapy is currently one of the most commonly used therapies in psoriasis and other skin diseases [4,11]. However, it should be emphasized that long-term skin irradiation with UV rays usually induces metabolic changes in pathological cells, e.g., in psoriatic plaques, however, it may also lead to inflammation and redox imbalance in normal cells surrounding skin lesions [7].

Due to the effects of UV radiation, it seems necessary to use compounds with antioxidant activity, aimed at countering the harmful effects of UV radiation, in particular on the keratinocytes of healthy skin. One such potential natural compound is Cannabidiol (CBD), a phytocannabinoid found in *Cannabis sativa* L., recently suggested as a possible pharmacotherapy approach to skin diseases [12,13]. It is well known that CBD does not have psychoactive properties [14], but exerts anti-inflammatory and antioxidant effects [15]. The antioxidant activity of CBD is linked to the targeted regulation of the redox state by maintaining the balance of the levels of oxidants and antioxidants [16]. CBD has been shown to trap ROS and also reduce their production by chelating transition metal ions [17]. CBD has also shown to increase the activity of major enzymatic antioxidants such as superoxide dismutase, thereby stimulating the metabolism of superoxide radicals in biological systems [18]. In addition, multiple doses of CBD administered to rats with chronic inflammation have been shown to cause increased activity of glutathione peroxidase and reductase [19]. The interaction of CBD with the immune system has also been shown to be involved in suppressing the body’s inflammatory response [20,21]. Given the above, it seems highly likely that CBD can prevent lipid alteration resulting from UV-induced oxidative stress and inflammatory processes. Most studies on the influence of UV radiation on the metabolism of phospholipids in keratinocytes are carried out on in vitro cultures [3,22,23], which do not fully reflect in vivo metabolic changes in keratinocytes in radiation-exposed skin. Additionally, several in vivo studies on the effects of CBD on the lipidomic profile especially in epidermal cells are very limited. Therefore, the aim of the present work is to explore changes in the lipid profile of keratinocytes in the skin of nude rats exposed to UVA and UVB radiation and treated topically with CBD.

## 2. Materials and Methods

### 2.1. Reagents/Chemicals

The internal standards of phospholipids and CERs were obtained from Avanti Polar Lipids, Inc. (Alabaster, AL, USA). All other chemicals were of the highest grade of purity commercially available; all solvents were with a degree of purity suitable for LC-MS. The water was Milli-Q purity (Advantage A10, Millipore Corporation, Billerica, MA, USA).

### 2.2. Animals and Experimental Design

Experiments were carried out on male nude rats (RH-FOXN1RNU) at the age of 8–9 weeks (body weight 260–302 g). Rats were kept under standardized conditions (12 h light/12 h dark cycles) and fed pellets containing a mixture of various dietary components such as proteins, fiber and minerals [24]. All experimental activities with rats were approved by the Local Ethics Committee for Animal Experiments in Olsztyn (Resolution No.37/2019 of 26 April 2019). The rats were divided into the following groups of six:

- Control group: rats were treated with non-toxic hydrophilic petrolatum applied topically on the back for 20 min every 12 h for 4 weeks;
- CBD group: rats were treated with CBD (2.5%; *w/w* in petrolatum) applied topically on the back for 20 min every 12 h for 4 weeks;
- UVA group: the skin of the backs of the rats was irradiated with UVA (365 nm, increasing dose from 0.5 to 5 J/cm^2^) every 48 h for 4 weeks;
- UVA + CBD group: the skin of the backs of the rats was irradiated with UVA every 48 h as in the UVA group and every 12 h, the backs of the rats were treated with CBD as in the CBD group;
- UVB group: the skin of the backs of the rats was irradiated with UVB (312 nm, increasing doses from 0.02 to 2 J/cm^2^) every 48 h for 4 weeks;
- UVB + CBD group: the skin of the backs of the rats was irradiated with UVB every 48 h as in to UVB group and every 12 h, the backs of the rats were additionally treated with CBD as in the CBD group.

The source of UV radiation was the lamp with UVA/UVB emitter (Cosmedico, Stuttgart, Germany) used in the treatment of human skin diseases. The constant distance (around 2 cm) between the irradiated skin and the radiator was ensured by the use of standard plastic combs, which allows maintaining a required constant dose of radiation over a specific time and protects the skin against burns and overheating.

At the end of the experiment, the animals were anesthetized with inhaled isoflurane and sacrificed by heart excision. The skin from the back of the animals was immediately placed into phosphate-buffered saline (PBS) and incubated on ice for 1 h. Next, samples were fragmented and then incubated overnight (4 °C) in dispase (1 mg/mL) to separate the layer of epidermal cells from the dermis. The epidermis was digested for 20 min with 2.5% trypsin to release keratinocytes clear fraction. Cells obtained after centrifugation (300× *g*, 3 min) were resuspended in PBS.

### 2.3. Lipidomic Analysis

#### 2.3.1. Lipid Extraction and Quantification of Phospholipid Content

The cell pellet was resuspended in 1 mL of Milli-Q water, and the total lipids were extracted using the Bligh and Dyer method [25]. The phospholipid amount in each lipid extract was determined with the phosphorus assay, carried out according to Bartlett and Lewis [26]. For the detailed experimental procedures of lipid extraction and phospholipid quantification, the reader is referred to a previously published study in which the same methods were used [22].

#### 2.3.2. UPLC-ESI-MS and MS/MS Analysis of Phospholipids

The separation of phospholipids was carried out by hydrophilic interaction liquid chromatography using an UPLC system (Agilent 1290; Agilent Technologies, Santa Clara, CA, USA) coupled with a QTOF mass spectrometer (Agilent 6540; Agilent Technologies, Santa Clara, CA, USA). HILIC-LC-MS was performed with an internal standard to confirm and quantify the ion variations observed in the spectra. The phospholipid standards used were phosphatidylcholines (PC)(14:0/14:0), lysophosphatidylcholines (LPC)(19:0), phosphatidylethanolamines (PE)(14:0/14:0), cardiolipin (CL)(14:0/14:0/14:0/14:0); phosphatidylinositol (PI)(16:0/16:0); phosphatidylserines (PS)(14:0/14:0). The solvent system consisted of mobile phase A [50% (*v*/*v*) acetonitrile, 25% (*v*/*v*) methanol, 25% (*v*/*v*) water with 1 mM ammonium acetate] and mobile phase B [55% (*v*/*v*) acetonitrile, 55% (*v*/*v*) methanol with 1 mM ammonium acetate]. The solvent gradient was set as follows: gradient started with 0% of mobile phase A, increased linearly to 100% of A in 20 min, held isocratically for 15 min, returning to initial conditions in 10 min. An amount of 25 μg of phospholipid extract contained in five μL of each sample was diluted in 10 μL of phospholipid standards mixture and 90 μL of eluent B was loaded into the Ascentis^®^ Si column (15 cm × 1 mm, 3 μm, Sigma-Aldrich). The mobile phase flow rate was 40 μL min^−1^. The QTOF mass spectrometer was operated in negative-ion mode. Typical ESI conditions were as follows: electrospray voltage, −3000 V; sheath gas flow, 13 L/min; capillary temperature, 250 °C. Data were collected in a data-dependent acquisition mode (DDA). The range of *m*/*z* 100–1500 was used to acquire parent ion scan spectra with the collision energy fixed at 35 eV. The LPE, PE, PI, PS, and CL species were analyzed as [M − H]^−^ ions, while LPC, PC and SM species were analyzed as acetate anions adducts [M + CH_3_COO]^−^. Data acquisition was carried out using the Mass Hunter data system (v B0.8.0, Agilent Technologies, Santa Clara, CA, USA). For normalization of the data, peak areas of the identified phospholipids were divided for the area of an internal standard corresponding to each class. The retention times and MS/MS spectra were used for validation of phospholipid species identification. Identified most abundant phospholipid species belonging to phosphatidylinositol (PI), cardiolipin (CL), PC, PE, PS, lyso-PE (LPE), LPC, and SM classes are presented in Supplementary Tables S1 and S2.

#### 2.3.3. RPLC-ESI-MS and MS/MS Analysis of CERs

The RP-LC-MS/MS was applied to obtain the CER profiles. The same Agilent UPLC-ESI-QTOF-MS system (Agilent 1290; Agilent 6540; Agilent Technologies, Santa Clara, CA, USA) was applied for analysis. Chromatographic separation of CERs was performed on RP-C18 column (Acquity BEH Shield 2.1 × 100 mm; 1.7 μm; Waters, Milford, MA, USA) in gradient elution with the mixture of water (20 mM ammonium formate, pH 5) and methanol. The QTOF mass spectrometer was operated in positive (electrospray voltage 3.5 kV) ion mode with a capillary temperature of 300 °C and a sheath gas flow of 8 L/min, as previously described in details [27].

#### 2.3.4. Data Processing

The filtering, peak detection, alignment and integration as well as the assignment of each phospholipid species was carried out by MZmine 2.30 software for obtained data [28].

#### 2.3.5. Statistical Analysis

Obtained results are expressed as the average ± standard deviation. Univariate and multivariate statistical analyses were performed using Metaboanalyst version 4.0 [29]. The sets of data obtained by MS/MS analysis were autoscaled and Principal component analysis (PCA) was performed. The Kruskal–Wallis test was used for univariate statistical analysis. A value of *p* < 0.05 was considered to be statistically significant. The heatmaps (Figure S1, in Supplementary Materials) was created using “Euclidean” as the clustering distance and “Ward” as the clustering algorithm.

### 2.4. Enzymatic Analysis

#### 2.4.1. Measurement of PLA_2_ Activity

The phospholipase A_2_ (PLA2-EC.3.1.1.4) activity was determined by spectrophotometric method using PLA2 Assay Kit (no. 765021, Cayman Chemical Company, Ann Arbor, MI, USA) according to the kit instructions [30]. The activity was calculated by measuring the absorbance at 414 nm, using the DTNB [5,5′-dithio-bis-(2-nitrobenzoic acid)] extinction coefficient of 10.66 per mM per cm, and reported as nmol/min/mg of cytosolic protein.

#### 2.4.2. Measurement of Neutral SMase Activity

The SMase activity was measured using a commercial kit from Sigma-Aldrich (no. MAK152, Sigma-Aldrich, St. Louis, MO, USA) according to the kit instructions. The activity was calculated by measuring the absorbance at 655 nm, using a standard curve created for colorimetric product. The activity was reported as mU/mg of cytosolic protein.

Protein level was quantified using the Bradford method [31].

#### 2.4.3. Statistical Analysis

Data are presented as mean values and their standard deviations (n = 6). One-way analysis of variance (ANOVA) with Bonferroni post-test was used for multiple comparisons to evaluate significant differences among groups. A value of *p* < 0.05 was considered to be statistically significant. Statistical analysis was performed using GraphPad Prism 7 software.

## 3. Results

In order to identify significant changes in the phospholipid profiles of experimental groups data obtained by LC-MS analysis were autoscaled and analyzed with the use of multivariate statistics. Principal component analysis (PCA), as an unsupervised method, was applied to get an overview of the clustering of the samples from each experimental group. Two sets of data containing four experimental groups were analyzed. Data from control groups exposed and not exposed to UVA radiation and/or treated with CBD (Control, CBD, UVA, UVA + CBD) were included in the first set. However, the second set of data was composed of the following groups: Control, CBD, UVB and UVB + CBD.

**Figure 1:**
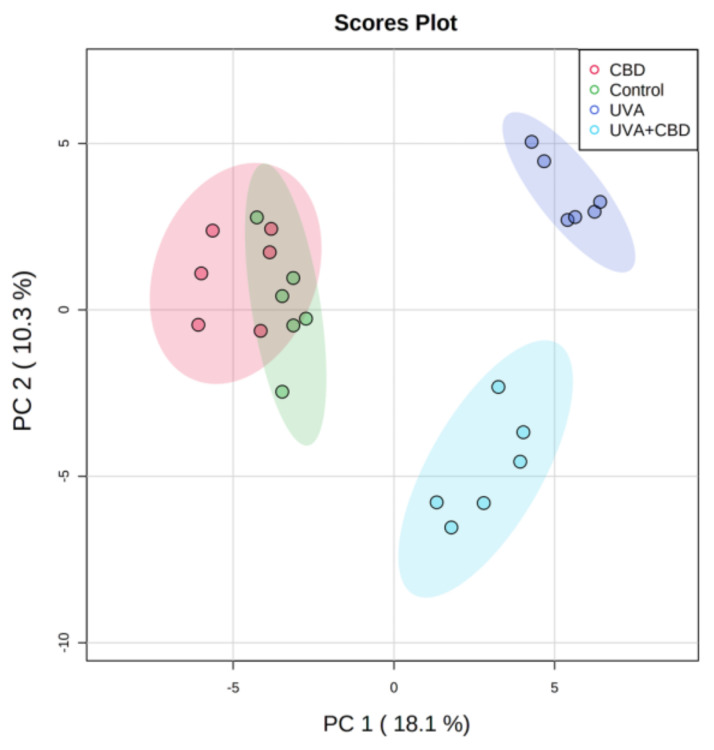
Two-dimensional principal component analysis (2D PCA) scores plots of the relative abundance of phospholipid species related to the internal standard of each phospholipid class in keratinocytes isolated from the skin of control (Control) and rats with UVA-irradiated rats (increasing doses of 0.5 to 5 J/cm^2^ for 4 weeks). These cells have not been treated (Control and UVA) or treated with cannabidiol (CBD) (CBD and UVA + CBD) (2.5 g CBD in 100 g petrolatum).

The two-dimensional principal component analysis (2D PCA) scores plot corresponding to the first data set shows that the model captured 28.4% of the total variance in the data set (Figure 1).

Principal component 1 (18.1%) was the major discriminating component responsible for the separation of control groups of rats (Control and CBD) from UVA irradiated groups, while component 2 (10.3%) describes the variation between two groups of rats irradiated with UVA (UVA and UVA + CBD). The 2D PCA plot shows that the group of rats irradiated with UVA was clearly separated from the group of animals irradiated with UVA and treated with CBD. However, the PCA model demonstrates that Control and CBD groups could not be clustered in a two-dimensional score plot. This indicates that the phospholipid profile did not significantly differ between these two groups (Figure 1).

**Figure 2:**
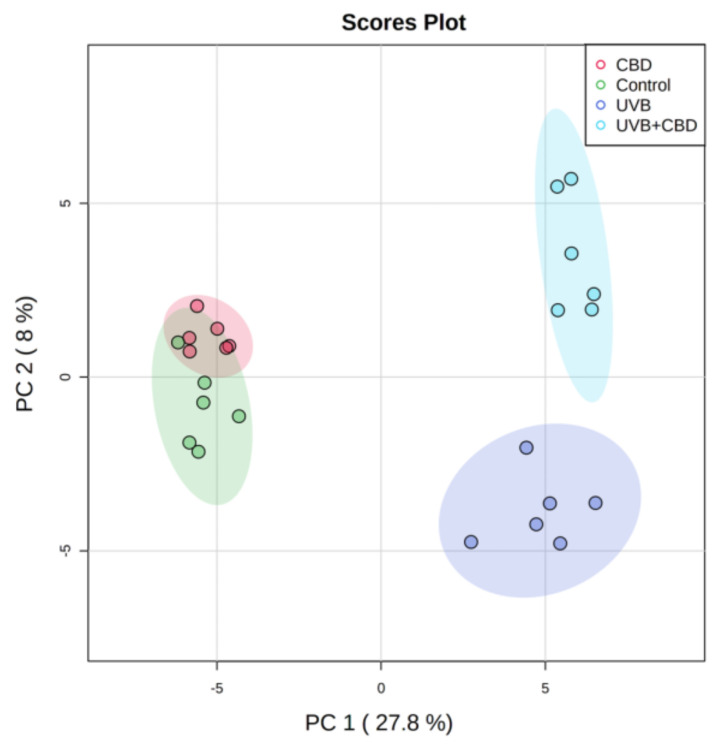
Two-dimensional principal component analysis (2D PCA) scores plots of the relative abundance of phospholipid species related to the internal standard of each phospholipid class in keratinocytes isolated from the skin of control (Control) and UVB-irradiated rats (increasing doses from 0.02 to 2 J/cm^2^ for 4 weeks). These cells have not been treated (Control and UVA) or treated with CBD (CBD and UVA + CBD) (2.5 g CBD in 100 g petrolatum).

The two-dimensional PCA score plot of the second set of data demonstrates the pattern where the rats irradiated with UVB were clearly separated from control rats treated and non-treated with CBD. The plot shows that the model describing 35.8% of the total variance including principal component 1 and principal component 2, which counts for 27.8% and 8%, respectively (Figure 2). Principal component 1, which represents the highest variation in the model, is responsible for observed discrimination between samples from rats exposed to UVB (UVB and UVB + CBD groups) and samples from non-irradiated rats (Control and CBD groups). Considering the poor separation of the control group from CBD, it seems that the phospholipid profile of keratinocytes from rats treated with CBD was similar to the profile of control rats. However, clustering of UVB and UVB + CBD groups suggests significant differences between phospholipid profiles of keratinocytes from animals belonging to these groups.

**Figure 3:**
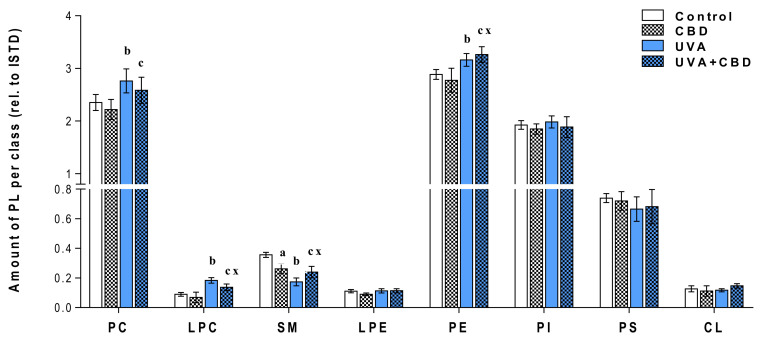
Changes in relative phospholipid content related to the internal standard of each phospholipid class within each class in keratinocytes isolated from the skin of control (Control) and UVA-irradiated rats (increasing doses from 0.5 to 5 J/cm^2^ for 4 weeks). These cells have not been treated (Control and UVA) or treated with CBD (CBD and UVA + CBD) (2.5 g CBD in 100 g petrolatum); values are mean ± SD, *p* < 0.05; (a), CBD vs. control; (b), UVA vs. control; (c), UVA + CBD vs. control; (x), UVA + CBD vs. UVA.

**Figure 4:**
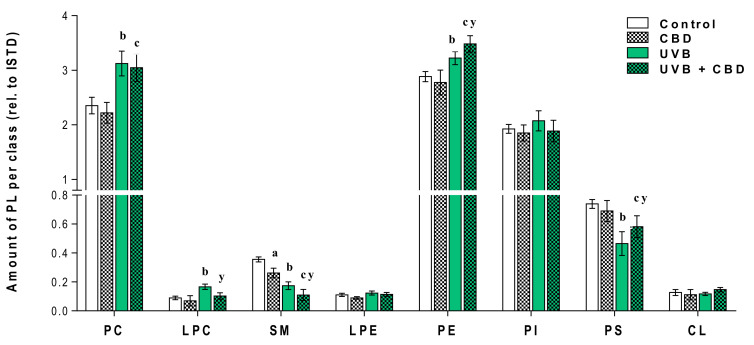
Changes in relative phospholipid content related to the internal standard of each phospholipid class within each class in keratinocytes isolated from the skin of control (Control) and UVB-irradiated rats (increasing doses from 0.02 to 2 J/cm^2^ for 4 weeks). These cells have not been treated (Control and UVB) or treated with CBD (CBD and UVB + CBD) (2.5 g CBD in 100 g petrolatum); values are mean ± SD, *p* < 0.05; (a), CBD vs. control; (b), UVB vs. control; (c), UVB + CBD vs. control; (y), UVB + CBD vs. UVB.

In the next step, we performed univariate analysis using the Kruskal–Wallis test following a post hoc Dunn multiple comparison test to assess the variation in the relative abundance of molecular species of phospholipids related to the internal standard of each phospholipid class under the conditions studied (Figure 3, Supplementary Tables S3 and S4, Figure S1). The same analysis was performed for the second set of data and the results corresponding to this set are shown in Figure 4 and Figure S1 and Table S4.

**Figure 5:**
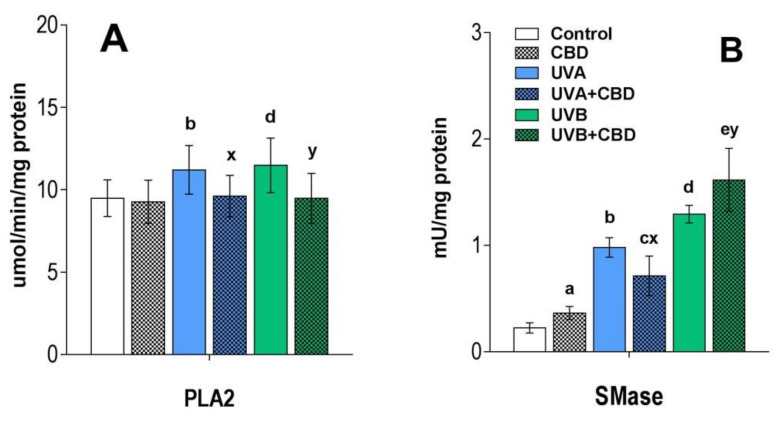
PLA2 activity panel (**A**) and neutral SMase activity panel (**B**) in keratinocytes isolated from the skin of control (Control) and UVA-irradiated rats (increasing doses from 0.5 to 5 J/cm^2^ for 4 weeks) or UVB (increasing doses from 0.02 to 2 J/cm^2^ for 4 weeks). These cells have not been treated (Control and UVA) or treated with CBD (CBD and UVA + CBD) (2.5 g CBD in 100 g petrolatum). The examined group of keratinocytes origin from two data sets each comprising four groups: (Control vs. CBD vs. UVA vs. UVA + CBD) and (Control vs. CBD vs. UVB vs. UVB + CBD). Values are mean ± SD, *p* < 0.05; (a), CBD vs. control; (b), UVA vs. control; (c), UVA + CBD vs. control; (d), UVB vs. control; (e), UVB + CBD vs. control; (x), UVA + CBD vs. UVA; (y), UVB + CBD vs. UVB.

Figure 3 and Figure 4 show that the changes induced by UVA and UVB radiation in the phospholipid profile of keratinocytes, namely the up-regulation of PC, LPC, PE and down-regulation of SM. The observed up-regulation of PE was similar in both rats irradiated with UVA and UVB, while an increase in the relative level of PC, as well as LPC, was more significant in the case of keratinocytes of rats chronically exposed to UVB radiation. Moreover, the observed increase in the relative content of LPC in the keratinocytes of the two groups of rats irradiated with different types of UV radiation (UVA or UVB) was accompanied by an increase in PLA2 activity (Figure 5).

In addition to the above changes, UVB radiation-induced a decrease in the relative level of PS, while in keratinocytes of rats chronically exposed to UVA, the level of PS was not significantly different from un-irradiated rats. We also observed a significant decrease in the level of SM in the keratinocytes of the skin of rats irradiated with UVA and UVB (Figure 3, Figure 4 and Figure S1), which was accompanied by an increase in the activity of SMase observed in these experimental groups (Figure 5).

**Figure 6:**
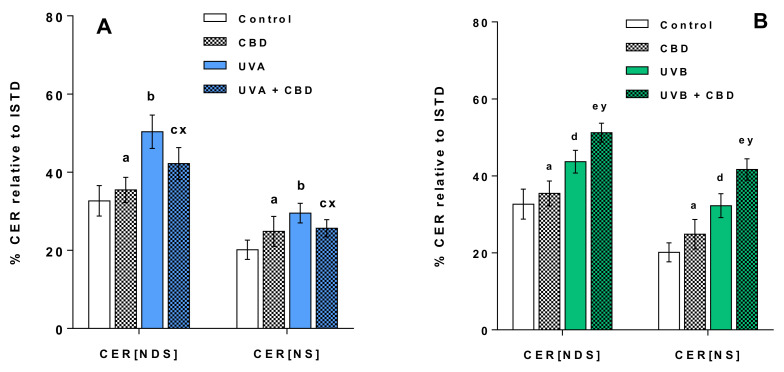
Changes in relative ceramide (CER) content related to the internal standard of each ceramide class within CER[NDS] and CER[NS] classes in keratinocytes isolated from the skin of control rats (Control) and rats irradiated with UVA (increasing doses from 0.5 to 5 J/cm^2^ for 4 weeks). These cells were not treated or treated with CBD (2.5 g CBD in 100 g petrolatum) panel (**A**); the skin of control rats (Control) and rats irradiated with UVB (increasing doses from 0.02 to 2 J/cm^2^ for 4 weeks). These cells were not treated or treated with CBD (2.5 g CBD in 100 g petrolatum) panel (**B**). The following groups of keratinocytes were examined: Control, CBD, UVA, UVA + CBD, UVB and UVB + CBD; values are mean ± SD, *p* < 0.05; (a), CBD vs. control; (b), UVA vs. control; (c), UVA + CBD vs. control; (d), UVB vs. control; (e), UVB + CBD vs. control; (x), UVA + CBD vs. UVA; (y), UVB + CBD vs. UVB.

We also identified the most abundant CER species belonging to two main classes of CERs, namely CERs containing non-hydroxy fatty acids and sphingosine (CER[NS]) and CERs containing non-hydroxy fatty acids and dihydrosphingosine (CER[NDS]), which represent the two main classes identified in keratinocytes (Supplementary Tables S5 and S6). We found that a decrease in the relative content of SM was accompanied by an up-regulation of CERs from the groups irradiated with UVA and UVB (Figure 6).

### Changes in the Lipid Profile of Rat Keratinocytes after Topical Application of CBD

The results obtained show that the topical application of CBD to the skin of rats irradiated with UVB led to a significant up-regulation of keratinocyte PS. In contrast to this, no change in PS content was observed in keratinocytes of rats irradiated with UVA after topical application of CBD (Figure 3 and Figure 4). In addition, the results indicate that long-term application of CBD to the skin of rats irradiated with UVA as well as UVB led to up-regulation of PE species. Our results also showed that while LPCs were up-regulated in keratinocytes from the skin of rats irradiated with UVA/UVB, topical application of CBD on the skin of these rats led to a significant down-regulation in these lysophospholipids, compared with UV treated cell. Moreover, the relative level of LPC in keratinocytes of UVB-irradiated rats treated with CBD was similar to that of control animals. The down-regulation observed in LPC in keratinocytes of rats exposed to UVA as well as UVB radiation was consistent with a decreased activity of PLA2 in these animals (Figure 5). We found that long-term application of CBD to the skin UVA/UVB-irradiated nude rats, as well as control animals, resulted in a decrease in the relative SM content (Figure 3 and Figure 4). The observed down-regulation of SM was accompanied by an increase in SMase activity (Figure 5). It should be emphasized that these changes in SM content and SMase activity were the only effects of topical application of CBD on the skin of un-irradiated rats.

The most discriminant species of phospholipids belonging to each class responsible for the changes described above are listed in Supplementary Tables S1 and S2 corresponding respectively to the first and to the second data set.

## 4. Discussion

### 4.1. Effects of UVA and UVB on the Lipid Profile of Rat Keratinocytes

The epidermis is a physiological barrier that protects the body against pathogens and chemical or physical factors, among which UV radiation is one of the most important that leads to impaired cell metabolism. Keratinocytes, the most abundant and exposed type of epidermal cell, absorb most UV radiation. However, changes in the lipid composition of keratinocytes resulting from exposure to UV radiation and the metabolic consequences of such an effect are still under investigation.

The main structural elements of the cell membrane responsible for its organization, but also cell signalling, are phospholipids. However, UV radiation, mainly through the induction of oxidative stress, leads to structural and functional changes of phospholipids [5,32,33]. Most of the studies on the effect of UV radiation on the metabolism of phospholipids in keratinocytes concern in vitro studies [3,22,23]. However, these changes do not fully correspond to the conditions in which all the skin is exposed to radiation. Therefore, the present study was performed to determine metabolic changes in skin keratinocytes of nude rats exposed to UVA and UVB radiation. The results obtained show that UVA and UVB induce changes in the phospholipid profile of keratinocytes, namely up-regulation of PC, LPC, PE and down-regulation of SM. Our previous in vitro study of UV irradiated keratinocytes also showed up-regulation of PC, plasmalogen phosphatidylcholine and ether bound phosphoethanolamine, while no significant changes were found in LPC [22]. The significant increase observed in this study of the relative content of LPC in the keratinocytes of the two groups of rats treated with different types of UV radiation (UVA or UVB), indicates an inflammatory process induced by the radiation. Lysophospholipids are generated by the action of PLA2, an enzyme that catalyses the hydrolysis of phospholipids in the sn-2 position, which confirmed the increased activity of keratinocytes of rats chronically irradiated with UVA or UVB in the present study. Previously, UV radiation has also been shown to significantly increase PLA2 activity in human keratinocytes cultured in vitro, which may explain the changes observed in the LPC content [34,35]. Furthermore, it is known that LPC can induce keratinocyte differentiation by activating protein kinase C which increases the expression of, e.g., transglutaminase-1, important proteins in this process [36,37]. The results of the present study also show that in the keratinocytes of rats irradiated with both types of UV, among the up-regulated PCs is the PC specie PC(34:1). The chemical structure of this phosphatidylcholine species PC(16:0/18:1) indicates the presence of palmitic acid (16:0) and oleic acid (18:1). It has been shown that PC(34:1), by activating genes encoding anti-inflammatory mediators, can regulate the cellular response to radiation-induced inflammation [38,39].

In addition to the above changes, UVB radiation induces a decrease in the relative level of PS, while in keratinocytes of rats chronically exposed to UVA, the level of PS is not significantly different from that of un-irradiated rat cells. The above changes may result from the fact that PS are very sensitive to oxidation by ROS [40], the overproduction of which is observed in keratinocytes exposed to UV radiation [3]. Data from the literature suggest that keratinocytes are more resistant to UVA than to UVB radiation [41]. This may explain the decrease in PS levels, observed only in rats irradiated with UVB. Moreover, UVB radiation is considered to be the causative agent of many of the effects attributed to UV causing DNA mutations and modification in gene expression [42]. However, the regulation of transcription is part of the cellular response to UV-induced stress and serves as a defence mechanism [10,43,44].

In this study, we also observed a significant decrease in the level of SM in the keratinocytes of the skin of rats irradiated with UVA and UVB, which is correlated with an increase in the activity of SMase. Neutral and acidic SMases have already been reported to be activated in human keratinocytes by UV radiation [3]. The reduction in the level of SM, probably associated with the SMase activity, suggests an increase in the level of CER since the degradation of SM by SMase is one of the main pathways of formation of CERs [45]. In support of this claim, we investigated changes in the levels of CERs, namely CER[NS] and CER[NDS], which represent two main classes of CERs identified in keratinocytes [27]. CER species belonged to these CER classes are involved in cell signaling and linked to cell proliferation, differentiation and apoptosis in the human epidermis [45]. Indeed, the results of the present study confirmed the up-regulation of CERs belonging to both classes, which is consistent with several previous studies indicating an increase in CER levels under UV radiation [23,46,47]. CERs are the main lipid species responsible for the permeation barrier function and therefore for preventing excessive water loss, which is one of the most important functions of the epidermal barrier. It has been suggested that the growth of CERs in the stratum corneum of humans due to UV radiation may be associated with the beneficial effects of phototherapy in skin diseases [48]. The results of this study showing the increased synthesis of CERs in keratinocytes of UV irradiated rat skin are consistent with previous studies, which showed that UVB and UVA radiation leads to increased synthesis of CERs [3].

Another important finding of our study is a significant increase in the level of PE in the keratinocytes of rats chronically exposed to UVA and UVB radiation. Previously, higher levels of PE have been shown to up-regulate autophagy, as part of a survival mechanism [49]. Although UV radiation is one of the factors inducing changes in phospholipid metabolism, our results show that exposure of keratinocytes to chronic UV radiation also induces an adaptive cellular response.

### 4.2. Changes in the Lipid Profile of Rat Keratinocytes after Topical Application of CBD

Since UV radiation intensifies oxidative stress, it consequently promotes the oxidative processes of cellular components, including phospholipids, leading to their structural and metabolic changes [5,7]. To counter these changes, it seems important to use an antioxidant compound. One of the potential natural antioxidants is CBD, the phytocannabinoid which has been shown to reduce oxidative stress and phospholipid modifications in vitro resulting from exposure of keratinocytes to UV radiation [50]. Unfortunately, the reported effects of CBD on UV irradiated keratinocytes cultured in vitro do not reflect the actual biological conditions. Therefore, this study verified the effectiveness of CBD in vivo by evaluating the changes in keratinocyte membrane phospholipids resulting from topical application of CBD to the skin of rats exposed to UV radiation.

The results of our study show that topical application of CBD to the skin of UVB-irradiated rats resulted in significant up-regulation of keratinocyte PS. In contrast, no change in PS content was observed in keratinocytes of rat skin irradiated with UVA and treated with CBD. The redox imbalance in apoptotic cells has been shown to promote the oxidation of PS by ROS, the overproduction of which accompanies the inflammatory processes induced by UV radiation [40]. The observed changes may suggest that CBD inhibits the oxidative modification of PS in keratinocytes of UVB-irradiated rats. Indeed, it has recently been shown that CBD partially prevents oxidative changes affecting phospholipids in vitro by reducing ROS levels and regulating the redox balance of keratinocytes [9,51]. PS participate in apoptosis and their translocation into the outer membrane layer is important for the recognition of apoptotic cells by macrophages [52].

Furthermore, our results show that long-term application of CBD to the skin of rats irradiated with UVA as well as UVB leads to up-regulation of PE species. Contrary to this finding, our recent study indicated that treatment with CBD of keratinocytes from healthy individuals cultured in vitro partially prevented an increase in the levels of PE resulting from exposure to UVB radiation [22]. However, since PE up-regulates autophagy, an increase in the PE content observed here may suggest that CBD promotes autophagy induced in keratinocytes by chronic UV irradiation, probably as a response to avoid cell death. Importantly, the induction of autophagy by CBD has already been reported [53].

Contrary to the observed up-regulation of PE, CBD reduced the elevated LPC level in rat skin keratinocytes after UV radiation to the level in control animals. These effects were not observed in in vitro studies on UVB-irradiated keratinocytes [22]. However, it has previously been reported that CBD can both stimulate and inhibit the activity of PLA2 depending on the conditions [54], but inhibition of PLA2 has been confirmed to improve the condition of the skin [55]. The reduction in PLA2 activity in UVA and UVB irradiated rat skin keratinocytes observed here may indicate the anti-inflammatory effect of CBD, as reported in the literature [50]. Therefore, our study showing a decrease in PLA2 activity in the rat keratinocytes exposed to UV radiation following the use of CBD may indicate the potential therapeutic value of this phytocannabinoid.

Regardless of the above findings, the results of this study revealed that long-term application of CBD to the skin of nude rats reduced the SM content of keratinocytes, which is consistent with the increased activity of SMase as determined in this study. It should be emphasized that these changes in SM content and SMase activity were the only effects of topical application of CBD on the skin of un-irradiated rats. Although no studies have been reported showing a direct effect of CBD on SMase, this cannabinoid has previously been shown to reduce the levels of SM in vivo [56].

However, depending on the type of UV radiation, we found different effects of topical application of CBD on the level of SM in rat keratinocytes. CBD led to up-regulation of SM in keratinocytes of UVA-irradiated rats, while SM was down-regulated in keratinocytes of rats exposed to UVB radiation. An opposite direction of changes in SM level is confirmed by changes in CER content, as well as SMase activity, which were down-regulated and up-regulated after UVA and UVB radiation, respectively. Therefore further studies are needed to explain the different observed effects of CBD on ceramide-sphingomyelin metabolism in keratinocytes after UVA and UVB irradiation.

## 5. Conclusions

This work shows that UVA and UVB radiation influences the enzyme-dependent and ROS-dependent metabolism of membrane phospholipids by increasing oxidative stress in rat keratinocytes, mainly with consequent up-regulation of LPC and PLA2 activity, and reduction of PS. In contrast, topical application of CBD to the skin of irradiated rats increases the level of PS which indicates the prevention of oxidative modification of phospholipids and levels of PE, which may suggest an increase in autophagy and, therefore, reduced effects of oxidative stress. Additionally, CBD, by activating SMase, up-regulates the metabolism of SM, which leads to increased generation of CERs. Consequently, CBD also improves the transepidermal barrier, preventing excessive water loss. Nonetheless, more research is needed to elucidate the exact mechanisms of action of CBD in the membrane phospholipids in vivo.

## Supplementary Materials

The following are available online at https://www.mdpi.com/2076-3921/9/12/1178/s1.

## Abbreviations

ANOVA	Analysis of variance
CBD	Cannabidiol
CER	Ceramide
CER[NDS]	Ceramides containing non-hydroxy fatty acids and dihydrosphingosine
CER[NS]	Ceramides containing non-hydroxy fatty acids and sphingosine
DDA	Data-dependent acquisition mode
DTNB	5,5′-dithio-bis-(2-nitrobenzoic acid)
ESI	Electrospray *ionization*
HILIC	Hydrophilic interaction liquid chromatography
UPLC	Ultra performance liquid chromatography
LPC	Lysophosphatidylcholine
LPE	Lysophosphoethanolamine
PBS	Phosphate buffered saline
PCA	Principal component analysis
PC	Phosphatidylcholine
PE	Phosphatidylethanolamine
PEo	Ether-linked phosphoethanolamine
PI	Phosphatidylinositol
PLA_2_	Phospholipase A_2_
PS	Phosphatidylserines
QTOF	Quadrupole time of flight mass spectrometer
ROS	Reactive oxygen species
RP	Reversed-phase
SM	Sphingomyelin
SMase	Sphingomyelinase
UPLC	Ultra performance liquid chromatography
