# Supplementary material for: Changes in Lipid Profile of Keratinocytes from Rat Skin Exposed to Chronic UVA or UVB Radiation and Topical Application of Cannabidiol
Source: Antioxidants (Basel). 2020 Nov 25;9(12):1178. doi: 10.3390/antiox9121178 (PMC7761402; doi:10.3390/antiox9121178)
Supplement: Supplementary file 1 [file antioxidants-09-01178-s001.zip › Captions for figures.docx]

**Figure S1.** Two-dimensional hierarchical clustering heat map of the main 25 phospholipid species (lowest p-values in Kruskal–Wallis analysis) identified in the keratinocytes isolated from the skin of: control rats (Control) and rats irradiated with UVA (increasing doses from 0.5 to 5 J/cm^2^ for 4 weeks). These cells were not treated or treated with CBD (2.5g CBD in 100g petrolatum). The following groups of keratinocytes were examined: Control, CBD, UVA, and UVA+CBD **(panel A)**; control rats (Control) and rats irradiated with UVB (increasing doses from 0.02 to 2 J/cm^2^ for 4 weeks). These cells were not treated or treated with CBD (2.5g CBD in 100g petrolatum). The following groups of keratinocytes were examined: Control, CBD, UVB, and UVB+CBD **(panel B).** The relative abundance of each species is indicated on the colour scale, with the figures indicating the difference in fold compared to the overall average. The dendrogram at the top represents the clustering of the sample groups. The dendrogram on the left represents the clustering of individual phospholipid species (relative to the change in relative abundance).
