# Supplementary material for: Changes in Lipid Profile of Keratinocytes from Rat Skin Exposed to Chronic UVA or UVB Radiation and Topical Application of Cannabidiol
Source: Antioxidants (Basel). 2020 Nov 25;9(12):1178. doi: 10.3390/antiox9121178 (PMC7761402; doi:10.3390/antiox9121178)

**Table S1**. Peak area of each phospholipid molecular species identified in the keratinocytes, isolated from the skin of control rats (Control) and rats irradiated with UVA (increasing doses from 0.5 to 5 J/cm^2^ for 4 weeks). These cells were not treated or treated with CBD (2.5g CBD in 100g petrolatum). Data obtained using MZmine software (XLSX)


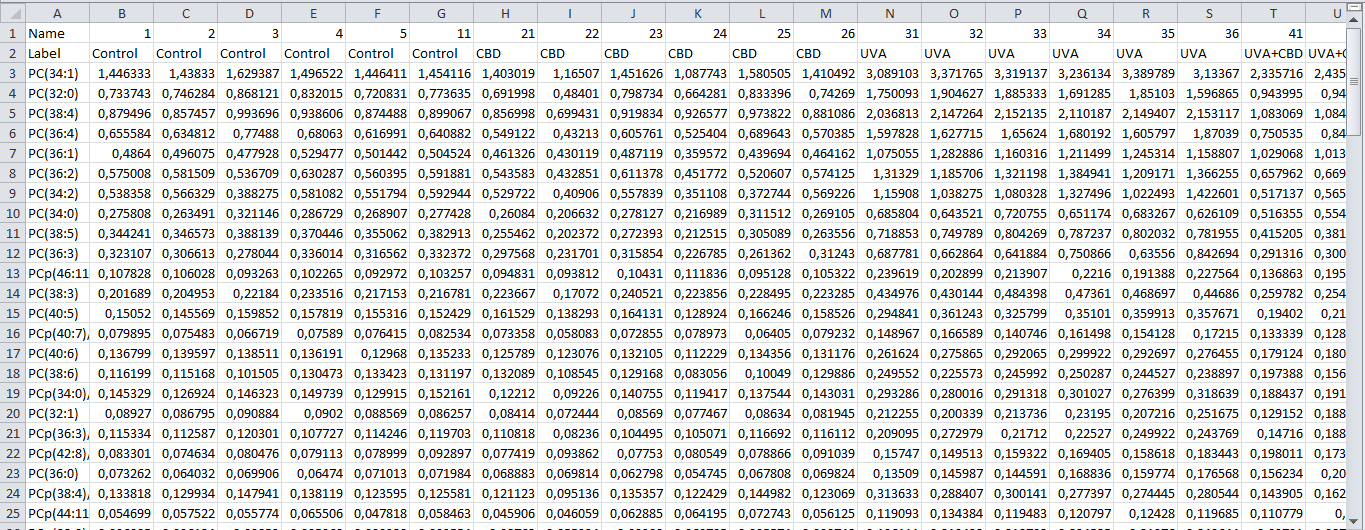


**Table S2**. Peak area of each phospholipid molecular species identified in the keratinocytes, isolated from the skin of control rats (Control) and rats irradiated with UVB (increasing doses from 0.02 to 2 J/cm^2^ for 4 weeks). These cells were not treated or treated with CBD (2.5g CBD in 100g petrolatum). Data obtained using MZmine software (XLSX).


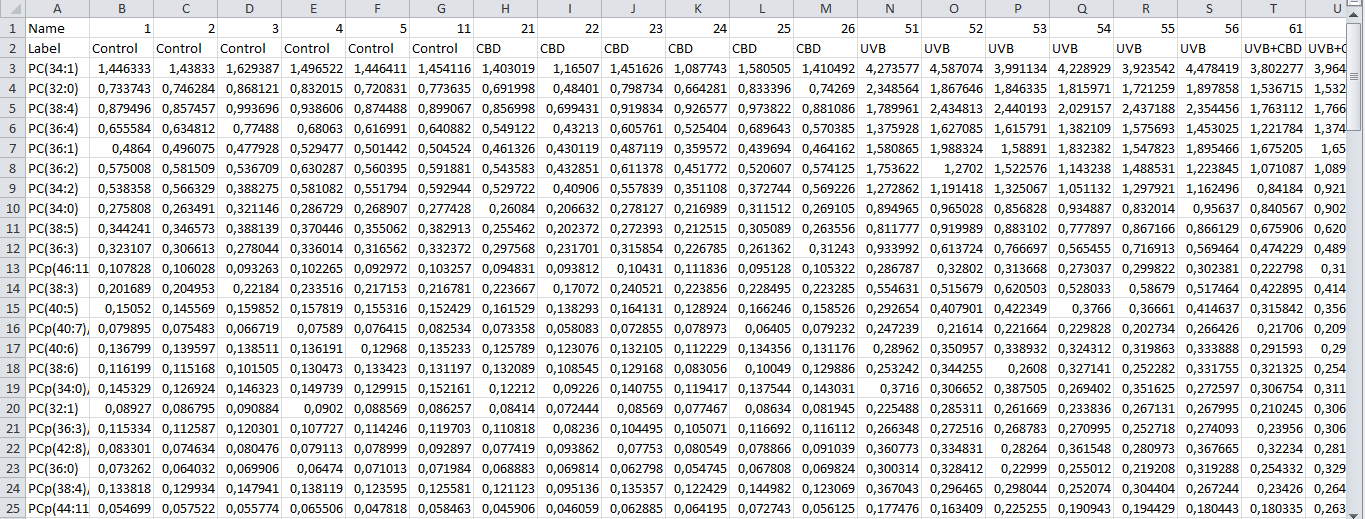


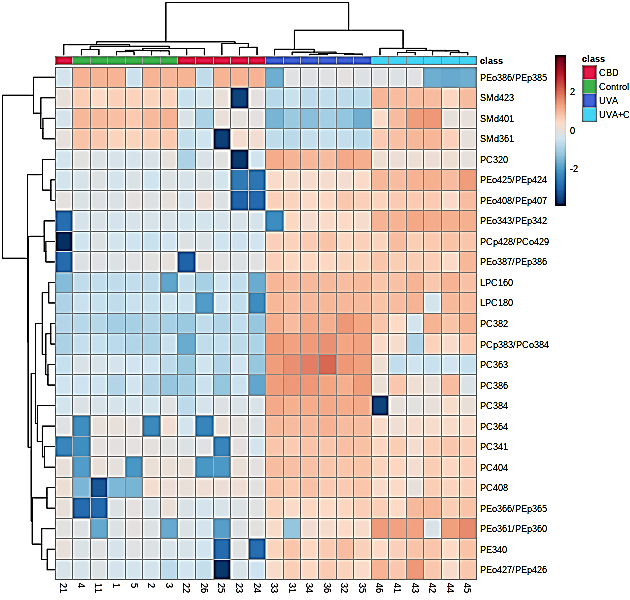

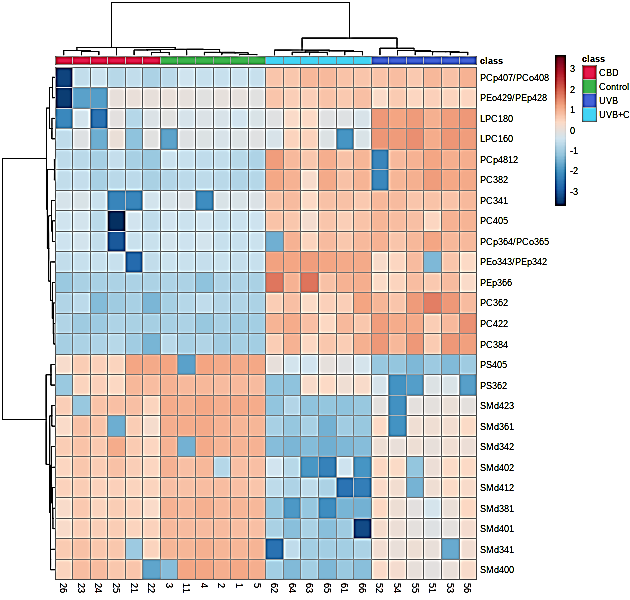


**A**

**B**

**Figure S1.** Two-dimensional hierarchical clustering heat map of the main 25 phospholipid species (lowest p-values in Kruskal–Wallis analysis) identified in the keratinocytes isolated from the skin of: control rats (Control) and rats irradiated with UVA (increasing doses from 0.5 to 5 J/cm^2^ for 4 weeks). These cells were not treated or treated with CBD (2.5g CBD in 100g petrolatum). The following groups of keratinocytes were examined: Control, CBD, UVA, and UVA+CBD **(panel A)**; control rats (Control) and rats irradiated with UVB (increasing doses from 0.02 to 2 J/cm^2^ for 4 weeks). These cells were not treated or treated with CBD (2.5g CBD in 100g petrolatum). The following groups of keratinocytes were examined: Control, CBD, UVB, and UVB+CBD **(panel B).** The relative abundance of each species is indicated on the colour scale, with the figures indicating the difference in fold compared to the overall average. The dendrogram at the top represents the clustering of the sample groups. The dendrogram on the left represents the clustering of individual phospholipid species (relative to the change in relative abundance).

**Table S3.** The alteration observed in the molecular species of most discriminating phospholipid species from PC, LPC, SM and PE class in the keratinocytes, isolated from the skin of control rats and rats treated with/or UVB and CBD, comparing CBD with control, UVA with control, UVA+CBD with control, UVA with UVA+CBD along with their respective fold change. All the alteration are significant at the P < 0.05 level. CBD, 2.5g CBD in 100g petrolatum; UVA, increasing doses from 0.5 to 5 J/cm^2^ for 4 weeks.

| **PL Class** | **Phospholipid specie** | **CBD vs control** | | **UVA vs control** | | **UVA+CBD vs control** | | **UVA+CBD vs UVA** | |
| --- | --- | --- | --- | --- | --- | --- | --- | --- | --- |
|  |  | **Adaptation** | **Fold change** | **Adaptation** | **Fold change** | **Adaptation** | **Fold change** | **Adaptation** | **Fold change** |
| PC | PC (36:3) |  |  | ↑ | 1.69 | ↑ | 0.73 |  |  |
|  | PC (38:2) |  |  | ↑ | 1.64 | ↑ | 0.75 |  |  |
|  | PC (38:6) |  |  | ↑ | 1.79 | ↑ | 0.68 |  |  |
|  | PC (40:8) |  |  | ↑ | 1.41 | ↑ | 0.64 |  |  |
|  | PC (32:0) |  |  | ↑ | 1.82 | ↑ | 0.72 |  |  |
|  | PC (34:1) |  |  | ↑ | 1.35 | ↑ | 0.77 |  |  |
|  | PC (38:4) |  |  | ↑ | 1.65 | ↑ | 0.69 |  |  |
|  | PC (40:4) |  |  | ↑ | 1.65 | ↑ | 0.74 |  |  |
|  | PC (36:4) |  |  | ↑ | 1.67 | ↑ | 0.61 |  |  |
|  | PCp(42:8)/PCo(42:9) |  |  | ↑ | 1.47 | ↑ | 0.72 |  |  |
|  | PCp(38:3)/PCo(38:4) |  |  | ↑ | 1.54 | ↑ | 0.54 |  |  |
| PE | PEo(42:5)/PEp(42:4) |  |  | ↑ | 1.43 | ↑ | 1.76 | ↑ | 0.22 |
|  | PEo(42:7)/PEp(42:6) |  |  | ↑ | 1.48 | ↑ | 1.73 | ↑ | 0.30 |
|  | PEo(36:1)/PEp(36:0) |  |  | ↑ | 1.32 | ↑ | 1.82 | ↑ | 0.17 |
|  | PEo(36:6)/PEp(36:5) |  |  | ↑ | 1.44 | ↑ | 1.77 | ↑ | 0.23 |
|  | PEo (34:3)/PEp(34:2) |  |  | ↑ | 1.34 | ↑ | 1.75 | ↑ | 0.27 |
|  | PEo (38:7)/PEp(38:6) |  |  | ↑ | 1.22 | ↑ | 1.71 | ↑ | 0.24 |
|  | PEo (40:8)/PEp(40:7) |  |  | ↑ | 1.28 | ↑ | 1.82 | ↑ | 0.31 |
|  | PE (34:0) |  |  | ↑ | 1.24 | ↑ | 1.66 | ↑ | 0.18 |
| LPC | LPC (16:0) |  |  | ↑ | 2.23 | ↑ | 1.62 | ↓ | 0.21 |
|  | LPC (18:0) |  |  | ↑ | 2.63 | ↑ | 1.54 | ↓ | 0.19 |
| SM | SM (d40:1) | ↓ | 0.78 | ↓ | 1.29 | ↓ | 0.92 | ↑ | 0.41 |
|  | SM (d42:3) | ↓ | 0.81 | ↓ | 1.34 | ↓ | 1.01 | ↑ | 0.33 |
|  | SM (d36:1) | ↓ | 0.72 | ↓ | 1.42 | ↓ | 0.86 | ↑ | 0.31 |

**Table S4.** The alteration observed in the molecular species of most discriminating phospholipid species from PC, LPC, SM, PE and PS class in the keratinocytes, isolated from the skin of control rats and rats treated with/or UVB and CBD, comparing CBD with control, UVB with control, UVB+CBD with control, UVB with UVB+CBD along with their respective fold change. All the alteration are significant at the P < 0.05 level. CBD, 2.5g CBD in 100g petrolatum; UVB, increasing doses from 0.02 to 2 J/cm^2^ for 4 weeks.

| **PL Class** | **Phospholipid specie** | **CBD vs control** | | **UVB vs control** | | **UVB+CBD vs control** | | **UVB+CBD vs UVB** | |
| --- | --- | --- | --- | --- | --- | --- | --- | --- | --- |
|  |  | **Adaptation** | **Fold change** | **Adaptation** | **Fold change** | **Adaptation** | **Fold change** | **Adaptation** | **Fold change** |
| PC | PC (34:1) |  |  | ↑ | 1.85 | ↑ | 0.84 |  |  |
|  | PC (38:4) |  |  | ↑ | 1.80 | ↑ | 0.82 |  |  |
|  | PC (42:2) |  |  | ↑ | 1.96 | ↑ | 0.89 |  |  |
|  | PC (40:5) |  |  | ↑ | 1.86 | ↑ | 0.70 |  |  |
|  | PC (38:2) |  |  | ↑ | 1.99 | ↑ | 0.91 |  |  |
|  | PC (36:2) |  |  | ↑ | 1.70 | ↑ | 0.67 |  |  |
|  | PCp(40:7)/PCo(40:8) |  |  | ↑ | 1.81 | ↑ | 0.82 |  |  |
|  | PCp(36:4)/PCo(36:5) |  |  | ↑ | 1.91 | ↑ | 0.85 |  |  |
| PE | PEo(44:12)/PEp(44:11) |  |  | ↑ | 1.67 | ↑ | 0.69 | ↑ | 0.28 |
|  | PEo(42:9)/PEp(42:8) |  |  | ↑ | 1.47 | ↑ | 0.72 | ↑ | 0.32 |
|  | PEo(34:3)/PEp(34:2) |  |  | ↑ | 1.54 | ↑ | 0.63 | ↑ | 0.19 |
|  | PEp(36:3) |  |  | ↑ | 1.43 | ↑ | 0.76 | ↑ | 0.21 |
| PS | PS (40:5) |  |  | ↓ | 1.48 | ↓ | 0.73 | ↑ | 0.31 |
|  | PS (36:2) |  |  | ↓ | 1.62 | ↓ | 0.69 | ↑ | 0.29 |
| LPC | LPC (16:0) |  |  | ↑ | 2.44 |  |  | ↓ | 1.56 |
|  | LPC (18:0) |  |  | ↑ | 3.21 |  |  | ↓ | 1.47 |
| SM | SM (d40:1) | ↓ | 0.76 | ↓ | 1.23 | ↓ | 1.97 | ↓ | 0.33 |
|  | SM (d42:3) | ↓ | 0.67 | ↓ | 1.32 | ↓ | 1.80 | ↓ | 0.17 |
|  | SM (d36:1) | ↓ | 0.58 | ↓ | 1.41 | ↓ | 1.78 | ↓ | 0.22 |
|  | SM (d34:2) | ↓ | 0.71 | ↓ | 1,36 | ↓ | 1.93 | ↓ | 0.30 |
|  | SM (d40:2) | ↓ | 0.54 | ↓ | 1.28 | ↓ | 1.84 | ↓ | 0.28 |
|  | SM (d38:1) | ↓ | 0.66 | ↓ | 1.44 | ↓ | 1.67 | ↓ | 0.22 |
|  | SM (d34:1) | ↓ | 0.57 | ↓ | 1.38 | ↓ | 1.89 | ↓ | 0.19 |
|  | SM (d40:0) | ↓ | 0.69 | ↓ | 1.65 | ↓ | 1.91 | ↓ | 0.32 |

**Table S5.** Peak area of each CER molecular species identified in the keratinocytes, isolated from the skin of control rats (Control) and rats irradiated with UVA (increasing doses from 0.5 to 5 J/cm^2^ for 4 weeks). These cells were not treated or treated with CBD (2.5g CBD in 100g petrolatum).


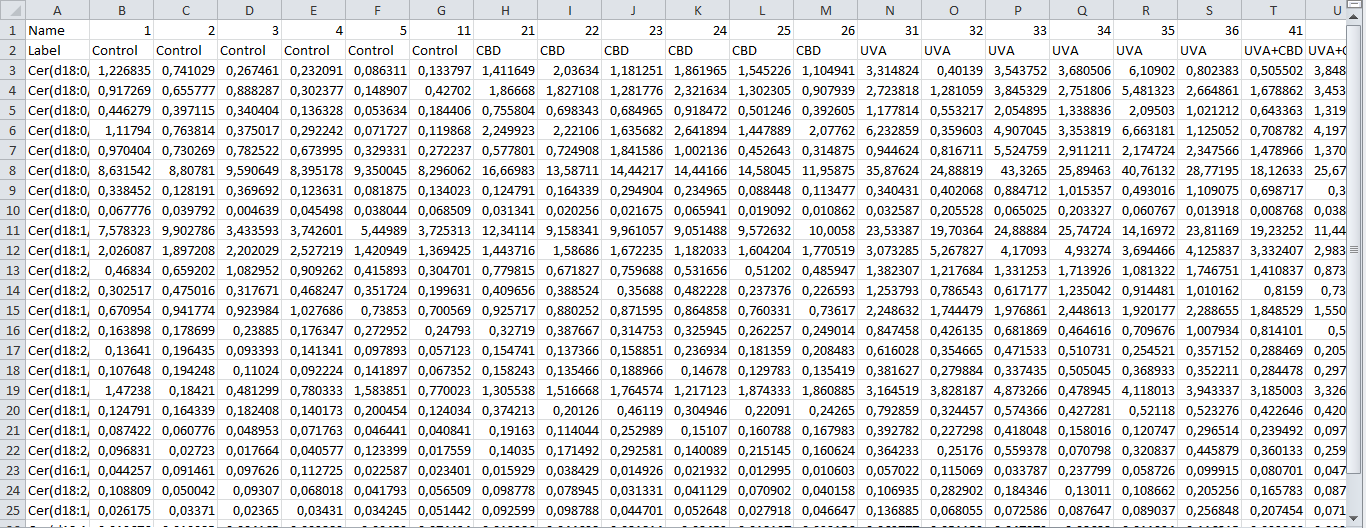


**Table S6.** Peak area of each CER molecular species identified in the keratinocytes, isolated from the skin of control rats (Control) and rats irradiated with UVB (increasing doses from 0.02 to 2 J/cm^2^ for 4 weeks). These cells were not treated or treated with CBD (2.5g CBD in 100g petrolatum). Data obtained using MZmine software (XLSX).


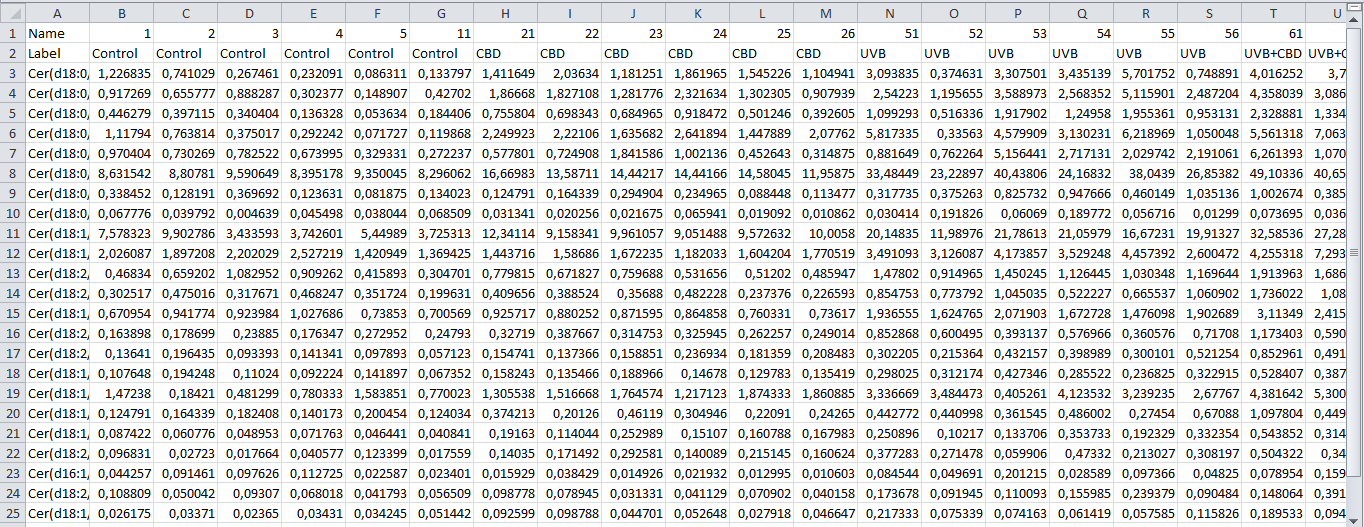

Supplement: Supplementary file 1 [file antioxidants-09-01178-s001.zip › Revised Supplementary materials.docx]
