# Supplementary figures and images for: Changes in Lipid Profile of Keratinocytes from Rat Skin Exposed to Chronic UVA or UVB Radiation and Topical Application of Cannabidiol
Source: Antioxidants (Basel). 2020 Nov 25;9(12):1178. doi: 10.3390/antiox9121178 (PMC7761402; doi:10.3390/antiox9121178)

A

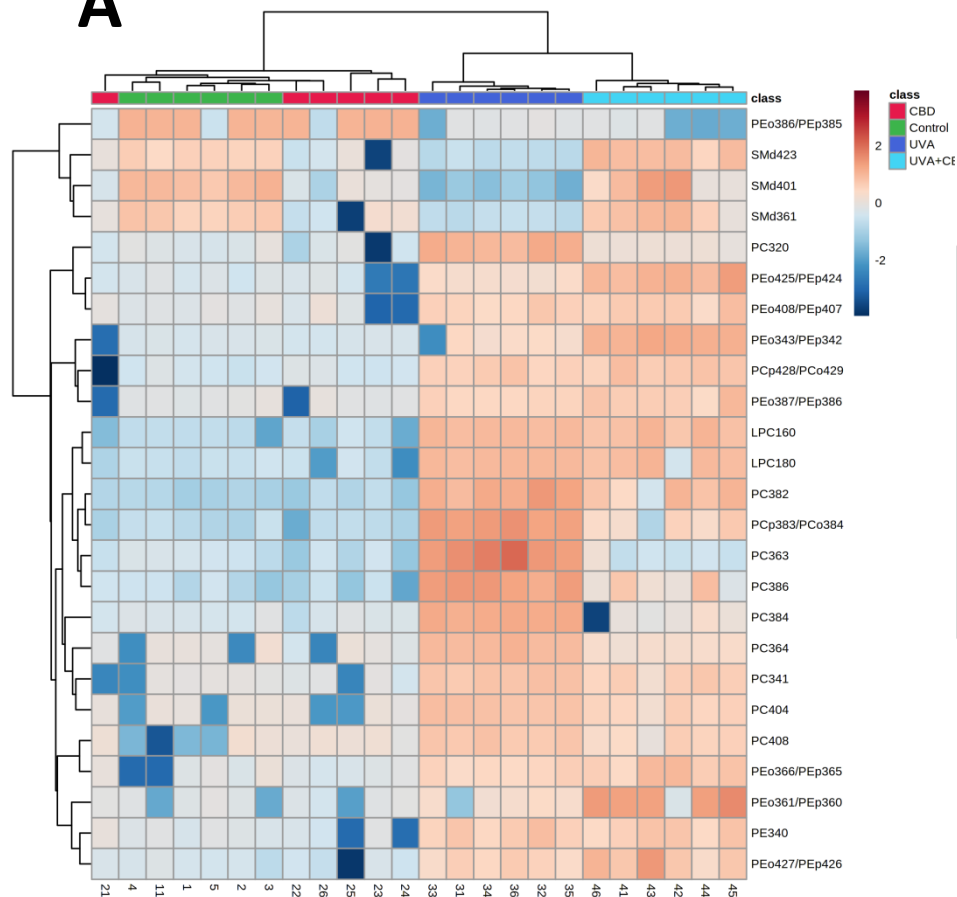

B

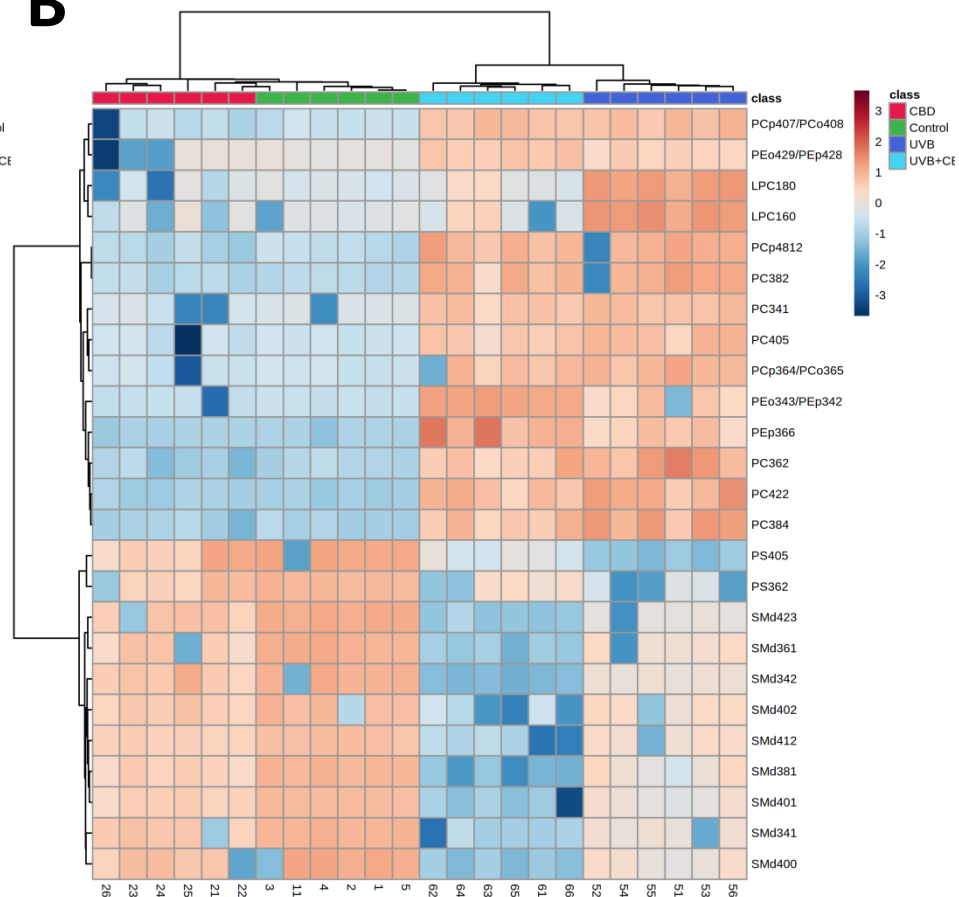

Supplement: Supplementary file 1 [file antioxidants-09-01178-s001.zip › Figure S1.pdf]
